# Identification and characterization of diverse groups of endogenous retroviruses in felids

**DOI:** 10.1186/s12977-015-0152-x

**Published:** 2015-03-15

**Authors:** Helena Mata, Jaime Gongora, Eduardo Eizirik, Brunna M Alves, Marcelo A Soares, Ana Paula Ravazzolo

**Affiliations:** Laboratório de Imunologia e Biologia Molecular, Faculdade de Veterinária, Universidade Federal do Rio Grande do Sul, Av. Bento Gonçalves 9090, CEP 91540-000 Porto Alegre, RS Brazil; Faculty of Veterinary Science, University of Sydney, Sydney, NSW 2006 Australia; Laboratório de Biologia Genômica e Molecular, Faculdade de Biociências, Pontifícia Universidade Católica do Rio Grande do Sul, Av. Ipiranga, 6681, CEP 90619-900 Porto Alegre, RS Brazil; Programa de Oncovirologia, Instituto Nacional de Câncer, Rua André Cavalcante, 37/4° andar, CEP 20231-050 Rio de Janeiro, RJ Brazil; Departamento de Genética, Universidade Federal do Rio de Janeiro, CCS – Bloco A, sala A2-120, Cidade Universitária, Ilha do Fundão, Rio de Janeiro, RJ Brazil

**Keywords:** Endogenous retroviruses, Felids, Neotropical cats, *pro/pol* region

## Abstract

**Background:**

Endogenous retroviruses (ERVs) are genetic elements with a retroviral origin that are integrated into vertebrate genomes. In felids (Mammalia, Carnivora, Felidae), ERVs have been described mostly in the domestic cat, and only rarely in wild species. To gain insight into the origins and evolutionary dynamics of endogenous retroviruses in felids, we have identified and characterized partial *pro/pol* ERV sequences from eight Neotropical wild cat species, belonging to three distinct lineages of Felidae. We also compared them with publicly available genomic sequences of *Felis catus* and *Panthera tigris*, as well as with representatives of other vertebrate groups, and performed phylogenetic and molecular dating analyses to investigate the pattern and timing of diversification of these retroviral elements.

**Results:**

We identified a high diversity of ERVs in the sampled felids, with a predominance of *Gammaretrovirus*-related sequences, including class I ERVs. Our data indicate that the identified ERVs arose from at least eleven horizontal interordinal transmissions from other mammals. Furthermore, we estimated that the majority of the *Gamma*-like integrations took place during the diversification of modern felids. Finally, our phylogenetic analyses indicate the presence of a genetically divergent group of sequences whose position in our phylogenetic tree was difficult to establish confidently relative to known retroviruses, and another lineage identified as ERVs belonging to class II.

**Conclusions:**

Retroviruses have circulated in felids along with their evolution. The majority of the deep clades of ERVs exist since the primary divergence of felids’ base and cluster with retroviruses of divergent mammalian lineages, suggesting horizontal interordinal transmission. Our findings highlight the importance of additional studies on the role of ERVs in the genome landscaping of other carnivore species.

**Electronic supplementary material:**

The online version of this article (doi:10.1186/s12977-015-0152-x) contains supplementary material, which is available to authorized users.

## Background

Endogenous retroviruses (ERVs) are remnants of exogenous counterparts that have integrated into the nuclear DNA of a germ-line cell of vertebrates and are transmitted through generations in a typical Mendelian fashion [1]. In the course of time, ERVs tend to become defective due to selection against functional retroviruses and also due to random mutations in the host genome [2,3]. Because they may have formed numerous copies in the host genome during or subsequent to the initial endogenization, behaving as transposable elements, ERVs can be represented by both mobile DNAs and remnants of ancient retroviral infections. ERVs and their exogenous counterparts are commonly grouped into three classes: Class I (ERV1, gammaretroviruses, and epsilonretroviruses), Class II (ERV2, alpharetroviruses, betaretoviruses, deltaretroviruses and lentiviruses) and Class III (ERV3 and spumaviruses) [3]. In addition to these groups, a new class of ERV (ERV4) has been described, with no characterised exogenous counterpart [4].

Endogenous retroviruses have been found in a wide variety of vertebrate species, including amphibians, reptiles, birds and mammals, as well as within the genomes of large DNA viruses, such as herpesviruses and poxviruses [5]. The potential of retroviruses to be horizontally transmitted between different species is shown by the close phylogenetic relationships inferred between viruses coming from distantly related hosts [6,7]. Among retroviruses, gammaretroviruses are able to infect a broad host range, contrasting with betaretroviruses that have a much narrower range [8]. Phylogenetic hypotheses bearing on retrovirus-host relationships are important because several pathogenic retroviruses arose after being transmitted between different hosts [5], and the understanding of retroviral evolution may help to monitor and/or minimize their potential impact.

Felids are an important animal model for some viral diseases offering strong parallels to related viruses in humans [9]. They can be infected by three known exogenous retroviruses, the feline immunodeficiency virus (FIV), the feline leukemia virus (FeLV) and the feline foamy virus (FFV). The former two are associated with serious illnesses, while FFV, like the human counterpart, causes no recognized disease. FIV induces an AIDS-like syndrome and is considered a model for HIV regarding lentiviral pathogenesis, therapeutic assays as well as for vaccine development. Therefore, the evolutionary history of *Retroviridae* in felids may help to clarify retrovirus evolution among mammals.

Endogenous retroviruses have been described mostly in the genome of the domestic cat, and only rarely in wild felids. Examples include the well-known endogenous feline leukemia virus (FeLV) and RD114 in *Felis*, *Beta*-like RV-domestic cat and RV-cougar [10], and the lineages FERV1-5 [11], FERVmlu1 and FERVmlu2 [12] and ERV-DC [13] in domestic cats. In a recent study, Song *et al*. [14] have described several novel ERVs in domestic cats, and currently nine divergent ERV class I lineages are known in these animals.

Felids comprise nearly 40 living species, eleven of which occur in the Neotropics and nine in Brazil, including the recently recognized *Leopardus guttulus* [15]. Neotropical cats belong to three of the major felid lineages, and one species, *Panthera onca*, is a member of the most basal extant group in the Felidae family [16]. In this context, if ERV lineages diversified during (or subsequent to) the divergence of the main felid groups, one might hypothesize that Neotropical cats, by representing three of the main felid clades, would carry distinct and possibly exclusive ERV lineages. In contrast, if the main episodes of ERV diversification happened prior to the divergence of the main felid groups, it could be hypothesized that Neotropical cats belonging to different clades would share some of the same ERV lineages, which would also be shared with cats from other geographic regions. Testing these alternative hypotheses would shed light on the evolutionary history of ERVs in felids and possibly in other mammals, given the potential for interspecies transmission involving cats and their prey. A related question is whether retroviruses integrated into the genome of wild cats frequently in the past, as shown for the domestic cat [14], or if these processes represent unusual events. In spite of their relevance, such tests have not been performed in Neotropical cats or across any other group of wild felids so far, due to the lack of available sequence data.

To address those questions, we experimentally identified novel endogenous retroviral sequences present in eight Neotropical cat species sampled in Brazil. Additionally, we searched for novel ERV sequences in available genome sequences from the domestic cat (*Felis catus*) and the Siberian tiger (*Panthera tigris altaica*). We used these data to perform phylogenetic and molecular dating analyses aiming to assess the evolutionary relationships of ERVs in Neotropical cats and those found in *Felis catus* and *Panthera tigris*, and to gain broader insights into the pattern and timing of diversification of endogenous retroviral elements in felids.

## Results

### Sequence overview

Eight Neotropical wild cat species (*P. concolor, P. yagouaroundi, L. geoffroyi, L. colocolo, L. guttulus, L. pardalis, L. wiedii* and *P. onca*) were screened by PCR and 85 ERV-like clones (out of 236) were identified, containing the typical retroviral PR-RT motifs and showing significant matches (e-values < 10^-10^) to ERV sequences deposited in GenBank. Out of 85 sequences, 80 were similar to class I ERVs, 4 similar to class II, and one was unclassified. The majority of these sequences (n = 82) were defective, presenting nonsense and frameshift mutations. Using these identified sequences as references, ERVs from the domestic cat (*Felis catus*), the Siberian tiger (*Panthera tigris altaica*) and several additional mammalian genomes were identified *in silico* (Additional file 1: Table S1).

**Figure 1:**
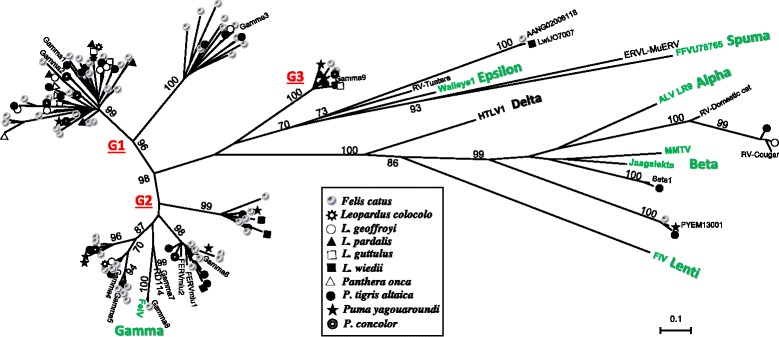
**Phylogenetic tree showing the diversity of felid endogenous retroviruses.** Maximum likelihood tree was based on deduced amino acid sequences of an RT fragment (Dataset1; 147 codons). Bootstrap values > 70% are indicated next to respective nodes (omitted for clarity on terminal branches). Host species’ designations are according to the inset graphical legend. G1 to G3 in red refer to distinct *Gammaretrovirus* groups identified based on the phylogenetic results and Gamma1-9 were previously described by Song *et al*. [14]. Exogenous retroviruses and their respective genera are in green. Retroviral sequences retrieved from GenBank are listed in Additional file 1: Table S1. The scale bar at the bottom represents the evolutionary distance in amino acid substitutions per site.

In the phylogenetic reconstruction using the RT fragment (Figure 1), the most abundant elements were represented by *Gammaretrovirus*-like (class I) sequences. Sequences belonging to class II ERVs and a divergent sequence (LwiJO7007), distantly related to the RV-Tuatara endogenous retrovirus [17], were also observed. Additionally, in a search for ERVs using *Censor* [18], a sequence from *L. wiedii* (LwiCT12006) showed 68% identity with ERV3-16A3_I, an ERV3-type (class III) endogenous retrovirus. This *L. wiedii* sequence was excluded from further analysis because it did not contain an identifiable RT domain. We did not find any sequences similar to exogenous retroviruses such as FIV and FFV among the 236 analyzed clones. Likewise, in Blastn searches against the *Panthera tigris* and *Felis catus* WGS database (as of October 2013) using *gag, pol* and *env* genes from FIV [GenBank: M25381.1] and from FFV [GenBank: Y08851.1] as query sequences, no ERV sequence presenting and *e*-value < 0.001 was retrieved in these genomes.

### Phylogenetic analysis of the divergent sequence from *Leopardus wiedii*

**Figure 2:**
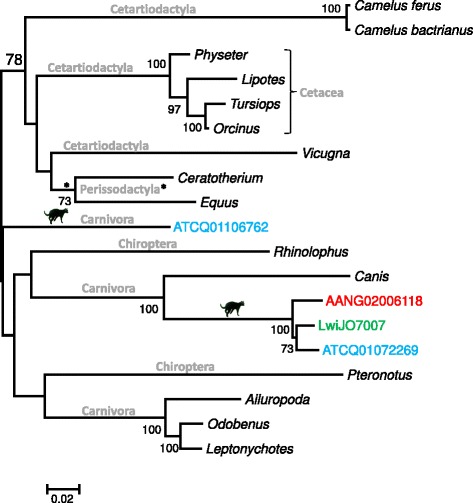
**Phylogenetic relationships among DNA sequences most closely related to LwiJO7007.** A maximum likelihood tree was constructed with a 613 bp-long alignment (Dataset 2). Bootstrap values > 70% are indicated next to their respective nodes. Mammalian orders containing sampled sequences are indicated above the branches defining each lineage. Felid illustrations are shown to designate their clades. Tiger is represented in blue, domestic cat in red and *L. wiedii* in green. The scale bar at the bottom represents distance in nucleotide substitutions per site. All sequences are listed in Table 1 and Additional file 1: Table S1.

The RT phylogeny depicted in Figure 1 shows the sequence of clone LwiJO7007 clustering closely with a sequence from the domestic cat, jointly forming a highly divergent group. A Blastn search using LwiJO7007 as a query sequence against the *Retroviridae* database did not retrieve any sequence similar to a known retrovirus. When using Blastx, the seven top hits corresponded to *Epsilonretrovirus* sequences, showing identities ranging between 31 and 34% (*e*-values < 10^-13^, queries covering > 92%). The Blastx search showed that 441 out of 641 nts belonged to the RT-like superfamily. A RepeatMasker analysis indicated that this retroelement is 67% identical to the shared segment of CarERV4-int, which was categorized as belonging to the ERV1 family (ERV Class I). In spite of its distant relationship to known/annotated ERVs, Blastn searches using LwiJO7007 against WGS databases revealed the existence of closely-related sequences (71-95% identity) in the genomes of domestic cat, tiger and several other mammals, encompassing four different placental orders (Figure 2 and Table 1). Sequences from specific PCR (LwiJO7007 group) showed that all species of Brazilian wild cats tested positive for this retroviral element (Additional file 2: Figure S1).

**Table 1:** **Sequences from mammalian hosts retrieved from Blast searches represented in Figure** **2** **(Dataset 2)**

**Species**	**Query coverage (%)**	***e*** **-value**	**Max identity**	**Accession #** ^**a**^	**Range** ^**b**^	**Order**
***Felis catus***	97	0	95	AANG02006118.1	1057-1676	Carnivora
***Panthera tigris altaica***	97	0	95	ATCQ01072269.1	24215-24856	Carnivora
***Panthera tigris altaica***	97	2e-97	74	ATCQ01106762.1	39362-39980	Carnivora
***Canis lupus familiaris***	93	9e-141	80	AACN010860585.1	176-777	Carnivora
***Odobenus rosmarus divergens***	96	4e-100	75	ANOP01031772.1	5018-5630	Carnivora
***Leptonychotes weddellii***	96	3e-102	75	APMU01047287.1	13176-13795	Carnivora
***Ailuropoda melanoleuca***	96	3e-86	74	ACTA01106447.1	76204-76825	Carnivora
***Mustela putorius furo***	94	6e-73	72	AGTQ01017509.1	71952-72251/72430-72511	Carnivora
***Equus caballus***	97	1e-107	75	AAWR02015524.1	24726-25088	Perissodactyla
***Ceratotherium simum simum***	97	9e-103	74	AKZM01043431.1	19498-20120	Perissodactyla
***Tursiops truncatus***	96	1e-81	72	ABRN02376857.1	9365-9980	Cetacea
***Rhinolophus ferrumequinum***	96	3e-90	74	AWHA01255713.1	24622-25239	Chiroptera
***Orcinus orca***	96	1e-82	72	ANOL02026629.1	13003-13615	Cetacea
***Lipotes vexillifer***	96	4e-81	72	AUPI01147203.1	71347-71960	Cetacea
***Camelus ferus***	96	1e-68	71	AGVR01045248.1	8938-9560	Artiodactyla
***Vicugna pacos***	97	3e-70	71	ABRR02069708.1	724-1354	Artiodactyla
***Physeter catodon***	96	3e-83	72	AWZP01009029.1	2974-3587	Cetacea
***Camelus bactrianus***	96	7e-66	71	CAOW010039523.1	1656-2278	Artiodactyla
***Pteronotus parnellii***	97	3e-71	71	AWGZ01108586.1	5963-6588	Chiroptera

^a^Sequences that were most similar to the LwiJO7007 element.

^b^Coordinates refer to the match sequence.

### In-depth phylogenetic analysis of *Gamma*-like ERVs

**Figure 3:**
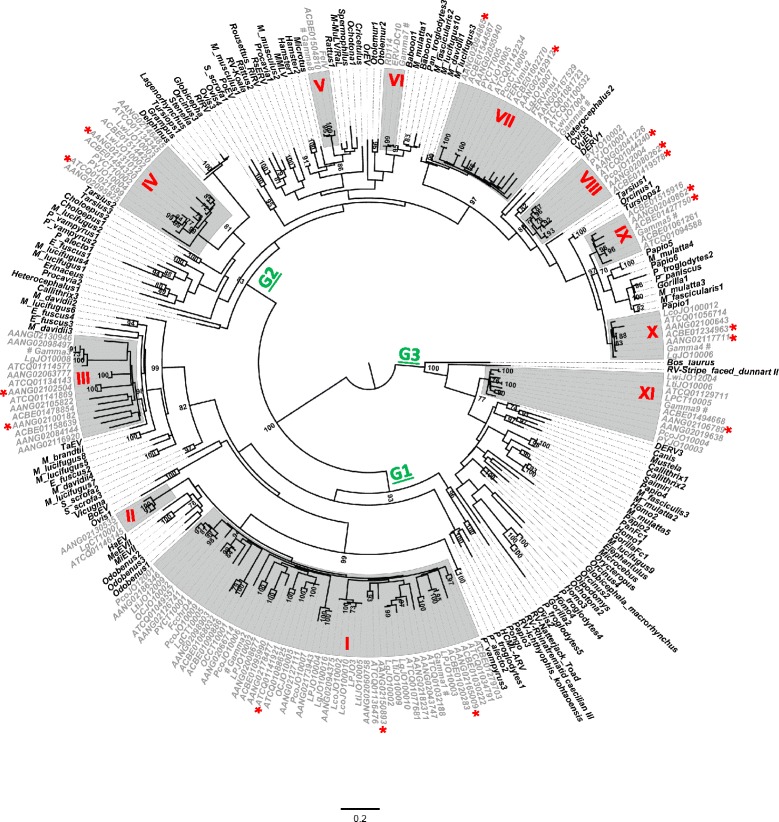
**Phylogenetic tree showing the diversity of felid gammaretroviruses.** A maximum likelihood tree was based on deduced amino acids of Pro-Pol fragments (Dataset 3; 198 codons). Bootstrap values > 70% are shown next to respective branches. The WGS sequences from *Panthera tigris altaica* [GenBank: ATCQ01000000], *Felis catus* [GenBank: AANG00000000] (Abyssinian breed) and [GenBank: ACBE00000000] (mixed breed) are indicated. *Puma concolor* (sequences starting with Pco)*, P. yagouaroundi* (Py)*, L. geoffroyi* (Lg)*, L. colocolo* (Lco)*, L. guttulus* (Lgu)*, L. pardalis* (LP)*, L. wiedii* (Lwi) and *P. onca* (OC) are also depicted. Roman numbers I to XI represent distinct *Gamma*-like retroviral lineages characterized in this study (marked in red). Asterisks indicate sequences mentioned in Table 2. The scale bar at the bottom of the Figure represents distance in amino acid substitutions per site. Sequences retrieved from GenBank are listed in Additional file 1: Table S1. ERVs termed Gamma1-9 by Song *et al*. [14] are indicated with the # symbol.

The phylogenetic analysis using Pro-Pol amino acid sequences (Figure 3) showed that *Gamma*-like sequences (including class I ERVs) from felids fall into three major phylogenetic groups (G1-G3), which were consistently supported by high bootstrap values. Interestingly, these clades also harboured sequences from diverse host species, representing several mammalian groups.

More specifically, we observed that the three major groups encompassed eleven distinguishable lineages of felid *Gamma*-like ERVs, most of which contained sequences from multiple cat species and were not obviously congruent with host phylogeny. The most diverse major group was G2, comprising seven felid ERV lineages, two of which correspond to the well-known FeLV and RD114. Groups G1 and G3 were less diverse, with the former containing three lineages and the latter containing a single lineage (see below). The sister groups of felids were very diverse, comprising sequences from other mammalian orders, such as cetartiodactylans (specifically cetaceans), primates, rodents, carnivores and bats, suggesting multiple separated transmission events among mammals (see Discussion).

### Characterization of the eleven *pol* gene lineages of class I ERVs from felids

A summary of the features of the *Gamma*-like lineages defined here (Figure 3) is provided in Table 2. Taking into account that *Felis catus* and *Panthera tigris* ERVs were present in almost all lineages, we were able to estimate approximate evolutionary ages of these groups by using LTR divergence within several proviral elements found in these genomes (see Methods). This age estimate was incorporated into the evolutionary characterization described below. However, these dates can only provide broad approximations considering that the LTR mutation rates from felid ERVs are unknown.

**Table 2:** **Information on** ***Gamma*** **-like lineages represented in Figure** **3**

	**Nonsense/frameshift mutations** ^**a**^	**Within group** ***p*** **-distance** ^**b**^ **(SE)**	**Description of lineage and time** ^**c**^	**Sequence ID** ^**d**^	**Contig length (bp)**	**Presumed ERV (positions within contig)**	**Avg .LTR length (bp)**	**LTR dist** ^**e**^ **/No. of differences** ^**f**^	**Time since integration** ^**g**^	**Time reported in the literature**
I	4 [1-10]	0.105 (0.005)	*Felis*, Pco, Py, Lco, Lgu, Lp, Lg, OC, tiger [10 MY]	AANG02165009.1AANG02150893.1 ATCQ01141921.1	12666 19424 35074	3998-12096 983-9298 24631-32736	166 294 298	0.28/27 0.131/32 0.084/22	60.8/28/11.7 28.5/13.1/5.5 18.26/8.4/3.5	gamma1 (30MY/8.2 MY/-) [14]
II	5 [4-6]	0.040	*Felis*, Lg, tiger [10 MY]	-	-	-	-	-	ND	-
III	11 [3- 14]	0.144 (0.006)	*Felis*, Lg, tiger [10 MY]	AANG02100182.1 AANG02102504.1 ATCQ01007049.1	91034 54996 22449	82322-90764 41175-50607 291-8884	343 260 314	0.20/53 0.22/43 0.20/49	43.7/20.1/8.4 49.1-22.6-9.4 45.2/20.8/8.7	
IV	2 [1-5]	0.093 (0.007)	*Felis*, Py, Lwi, tiger [10 MY]	AANG02131012.1 ATCQ01021699.1	6326 12087	280-6325 2033-9148	341 398	0.024/08 0.043/16	5.2/2.4/1 9.35/4.3/1.8	
V	0 [0]	0.113	*Felis* [3.36 MY]	enFeLV-AGTT (AY364318.1)	9895	9895	568	0/0	Recent [45]	
VI	0 [0-3]	0.037	*Felis* [3.36 MY]	ERV-DC10 (AB674444.1)	9363	9363	551	0/0	Recent [13]	The oldest ERV-DC integration (2.8 MY) [13]
VII	3 [0-6]	0.054 (0.004)	*Felis*, Py, Pco, Lwi, tiger [10 MY]	AANG02094865.1AANG02162912.1	23420 80539	4845-11356 2477-9063	336 375	0.064/19 0.028/10	13.9/6.4/2.7 6.1/2.8/1.2	gamma 6 (16 to 3.8 MY) [14]
VIII	2 [0-4]	0.046 (0.005)	*Felis*, Pco, Py, tiger [10 MY]	ACBE01419578.1 AANG02080262.1ATCQ01044240.1	12392 23123 16168	2915-9700 2586-11074 5551-14181	542 538 501	0.069/34 0.042/21 0.029/14	15/6.9/2.9 9.3/4.2/1.75 6.3-2.9-1.21	
IX	1 [0-5]	0.058 (0.005)	*Felis*, tiger [10 MY]	AANG02049862.1ACBE01427750.1	44805 11784	19379-27642 10858-2531	381 489	0.046/16 0.063/22	10/4.6/1.9 13.7/6.3/2.6	
X	0 [0-4]	0.043 (0.004)	*Felis*, Lg, Lco, tiger [10 MY]	ACBE01234963.1 AANG02117711.1	12498 14995	2151-9706 4735-14409	632 626	0.023/14 0.013/08	5/2.3/0.96 2.8/1.3/0.54	
XI	2 [0-4]	0.072	*Felis*, Py, Lgu, Lwi, LP, Pco, tiger [10 MY]	AANG02106789.1	17944	1- 5793	348	0.086/26	18.7/8.6/3.6	

^a^Median [min-max] values.

^b^Nucleotide distances within lineages.

^c^Time based on Johnson et al. [16]. The abbreviations of species’ names in column 4 are as in the legend of Figure 3.

^d^Only representatives of full-length ERVs from the domestic cat and tiger genomes are in column 5.

^e^Genetic distances (Kimura 2 parameter model) between LTRs.

^f^Number of differences between the 5′ and 3′LTRs.

^g^The three depicted values are based on mutation rates of 2.3e-9, 5e-9 and 1.2e-8 substitutions/site/year (see text for details), respectively. MY, million years; ND, not dated.

*Lineage I* is the most diverse group represented in the tree, comprising sequences of all species studied (experimentally and *in silico*), except for *Leopardus wiedii.* Additionally, our lineage-specific PCR recovered a sequence of *L. wiedii* 95% (e-value 3E-54) similar to ERV gamma1 [14] through Blastn (Additional file 3: Table S2). This lineage also presents a high intragroup diversity (0.1; Table 2). The [GenBank: AANG02165009.1] sequence from the domestic cat (see Table 2) represents a very old integration event (with its age estimated between 11.7 and 60.8 million years (MY) falling into the Miocene or even earlier), while the [GenBank: ATCQ01141921.1] sequence from the Siberian tiger was inferred to be much younger, ranging between 18 and 3.5 MY (Miocene-Pliocene), with a high degree of *pro*-*pol* intactness, presenting only three nonsense/frameshift mutations.

In contrast, *lineage II* is the least diverse clade represented in the phylogenetic tree and only harbors sequences from *L. geoffroyi*, Siberian tiger and domestic cat in our first analysis. The lineage-specific PCR demonstrated that lineage II is a genomic component of all species studied (Additional file 3: Table S2, Additional file 4: Figure S2). In the Megablast search using the LgCT10007 sequence as query, we retrieved only three hits out of 125 with similarity > 85% and coverage > 10% which belong to *Panthera tigris* [GenBank: ATCQ01146145.1], similarity of 97%, *e*-value 0.0) and *Felis catus* ([GenBank: AANG02130535.1] - Abyssinian breed - and [GenBank: ACBE01580426.1]) - mixed breed, both presenting similarity of 95% and *e*-value of 0.0). It was not possible to recover both LTRs from the sequences mentioned above, which prevented the estimation of their time of insertion in the genome. Nonetheless, we observed that this lineage is related (albeit with low support) to sequences from the seal *Halichoerus grypus* (HaEV) and the walrus *Odobenus rosmarus divergens*, both of which are marine carnivores (pinnipeds), as well as to *Meles meles* (MeEVII) and *Mustela vison* (MiEVII), both mustelids.

*Lineage III* is closely related to sequences from bats and presents the highest intragroup distance analyzed in this study (0.144; Table 2). This lineage also displays the lowest degree of *pro-pol* intactness, as evaluated by nonsense/frameshift mutations (median of 11). Accordingly, LTR divergences of three sequences indicated very ancient insertion events (estimated between 8 and 49 MY falling into Miocene-Oligocene). Furthermore, the longer branch lengths in the tree demonstrate that this lineage has persisted in the host genomes for long periods. Lineage-specific PCR confirm that this lineage is present in the genome of all species analysed (Additional file 4: Figure S2).

*Lineage IV* is related to ERVs from cetaceans, but separated from them by a long branch. ERVs from this lineage are present inside the genome of all species of Brazilian wild cat (Additional file 3: Table S2, Additional file 4: Figure S2). The two sequences of this lineage exemplified in Table 2 show recent insertion times compared to others (from 9 to 1 MY), ranging from the end of Miocene into the Pleistocene). It is noteworthy that this lineage presents few nonsense/frameshift mutations (maximum of 5) and a relatively high degree of *pro*-*pol* intactness.

*Lineage V* (FeLV lineage) and *lineage VI* (RD114/ERV-DC) are well-known ERVs present only in felids from the domestic cat lineage (*Felis*). The samples from the South American cats and the Blast searches of the Siberian tiger genome did not reveal any sequences related to these lineages, confirming previous studies (see [13,19]).

*Lineage VII* is closely related to sequences from bats, while *lineage VIII* is related to canine sequences (DERV1 and VuEV). *Lineage IX* is related to sequences from primates and *lineage X* is basal to lineage IX and to the primate clade. These lineages show a very similar pattern, with low rates of intragroup distance and few nonsense/frameshift mutations when compared to the others, and they are also a genomic component of all species examined in this study (Additional file 3: Table S2, Additional file 4: Figure S2). Dating based on LTR divergence showed that the insertion of some ERV sequences ranged from the middle Miocene to the early Pleistocene (see Table 2), near the time of diversification of modern felids [16]. The short branch lengths generally observed in the topologies (Figure 3, lineages VII to X) further support the inference of rapid diversification. Moreover, we detected short sequences by PCR (~325 bp of RT domain) from *L. pardalis* and *L. colocolo* that clustered within lineage VII in a ML tree (not shown), indicating that this lineage is very abundant in felid genomes.

*Lineage XI* is the only felid representative of the G3 group, inferred to be a sister clade to sequences from several other mammalian orders. Similar to the lineages described above, this fossil retrovirus is found in all species examined (Additional file 3: Table S2, Additional file 4: Figure S2). The LTR divergence of a sequence from domestic cat shows an insertion event ranging from Miocene (18.7 MY) to Pliocene (3.6 MY). The short branch lengths observed in the topology (Figure 3), the low intragroup diversity and the high degree of *pro-pol* intactness (Table 2) all indicate a recent diversification of this group.

### Phylogenetic analysis of felid ERV class II sequences

**Figure 4:**
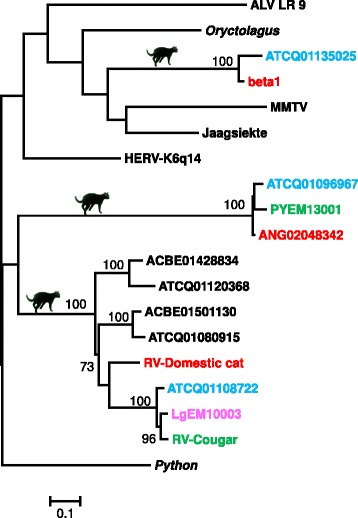
**Phylogenetic tree showing the relationships of class II ERV sequences.** A maximum likelihood tree was based on deduced amino acids of Pro-Pol fragments (Dataset 4; 236 codons). Bootstrap values >70% are indicated. Felid illustrations are shown to designate their clades. The three ERV clades mentioned in the text are highlighted by showing sequences from four of the major felid lineages: blue (from tiger belonging to the *Panthera* lineage), red (cat, domestic cat lineage), green (PYEM13001, from *P. yagouaroundi* and *Puma concolor*, both from the *Puma* lineage) and pink (*L. geoffroyi,* ocelot lineage). The scale bar at the bottom of the Figure represents distance in amino acid substitutions per site. Sequences retrieved from GenBank are listed in Additional file 1: Table S1.

We have also identified ERVs belonging to class II (Dataset 4; Figure 4), by using our sequences and others previously described as queries in Blast searches. This group seems to be less diverse than class I (*Gamma*-like) sequences. We found 12 Megablast hits with score > 200 using LgEM10003 as query against Felidae (taxid: 9681) and the same number of hits by using PyEM13001. This contrasted, for example, with the use of *Gamma*-like PyJO10001 sequence as query, when we found almost a two-fold number of Megablast hits (20 with score > 200).

Class II ERVs from felids grouped into three different clades which were well supported by bootstrap values (Figure 4). One group was formed by the *Betaretrovirus*-like viruses previously described [14]. The second was described by Gifford *et al*. [10], and also included a sequence from *Leopardus geoffroyi* retrieved by PCR. The third clade is newly identified in this study, which grouped with a sequence from *P. yagouaroundi*. According to our phylogenetic estimates (Figures 1 and 4), this group is basal to exogenous alpha- and betaretroviruses, but there was no statistical support for this resolution. As a whole, we could observe that class II ERVs are present in at least five different felid species, belonging to four different evolutionary lineages within Felidae (Figure 4).

## Discussion

This study represents the first broad assessment of the genome landscape of ERVs in the Felidae family, since similar assessments reported previously were restricted to the domestic cat [14]. Our study initially focused on the characterization of ERV sequences of Neotropical wild cats, and was expanded by further analyzing ERV sequences retrieved from the genomes *Felis catus* and *Panthera tigris*, thus unveiling general patterns of ERV diversity across the Felidae family. Firstly, our results indicate that different class I ERV lineages from felids are generally not congruent with their currently accepted host phylogenies (see Figure 3), possibly due to lack of resolution given the short segments analysed, or to variable sorting of ancestral polymorphisms. Furthermore, ERV sequences from Neotropical cats did not form a unique (monophyletic) clade, which could be expected from more recent infection events, for instance after they colonized South America. In contrast, phylogenetic reconstructions showed well supported ERV clusters from felids dispersed across the retroviral phylogenies, all presenting sequences of *Felis catus* and *Panthera tigris*, except for the RD114, FeLV and ERV-DCs gammaretroviruses, which are found only in *Felis*, but not in other closely related Felidae genera [13,19]. Secondly, in a recent study using only domestic cat samples, Song *et al*. [14] obtained a similar pattern on class I ERV diversity to the one shown here. Additionally, Cho *et al*. [20] showed that the cat and tiger genomes have very similar composition and ratios of repeat elements. However, it is unlikely that the present study exhausted the diversity of ERVs in Neotropical cats, considering the numerous retroviral sequences found during our searches using WGS of *Felis catus* and *Panthera tigris*. We expect additional, distinct lineages to be yet undiscovered.

We found sequences that could be classified as class II ERVs in felid genomes, but as expected we did not find any sequences similar to exogenous feline immunodeficiency viruses (FIV). Many retroviruses do not infect germ line cells, and the majority of integration events do not become fixed [21]. In fact, no endogenous copy of FIV has been found so far, although some FIV strains found in felids are deeply divergent, consistent with a long permanence, and may have existed within the Felidae family since the late Pliocene [22].

We failed to recover class III ERVs (spumaviruses) in the analysed felids, except for one sequence from *L. wiedii*. However, Song *et al*. [14] reported that class III ERVs are very abundant in *Felis catus*, although these *in silico* identified sequences are very divergent to group with other retroviral sequences, such as the sequence from *L. wiedii*. Therefore, class III ERVs in felids are probably more divergent than class I and II elements, which may have contributed to our failure in retrieving them by PCR.

Our phylogenetic analyses provided evidence of two divergent ERV sequence groups in felids. The most divergent group is related to clone LwiJO7007 from *Leopardus wiedii*, whose position in our phylogenetic tree was difficult to establish confidently relative to known retroviruses possibly because we did not detect a longer proviral sequence to improve the resolution of our analyses. However, our results strongly indicate that it is related to a group of sequences that colonized multiple mammalian genomes, as they have also been detected in several species (see Figure 2). These ERVs are very ancient components of Felidae genomes since the topological resolutions of a tree reconstructed using sequences from our lineage-specific PCR strategy (Additional file 2: Figure S1) resembles a phylogenetic split at species-level. The isolated position of a sequence from *Panthera tigris* [GenBank: ATCQ01106762] relative to the other felids in Figure 2 suggests that felid genomes have been colonized at least twice by this retroelement.

The other divergent group is formed by sequences related to PyEM13001 from *P. yagouaroundi*, and grouped with class II ERVs in our analyses. This clade seems to be intermediate between alpha- and betaretroviruses, perhaps being categorized provisionally as an alphabeta group, as named by Bolisetty *et al.* in a study on avian ERVs [23]. Further studies are thus warranted to better characterize and understand the evolutionary relationships within these two divergent groups and also their positioning among retroviruses.

An important motivation for more detailed analysis of *Gamma*-like ERVs was to gain some insight into viruses that infected wild cats in an ancient retroviral world. By means of phylogenetic reconstruction, we provide a broader evolutionary spectrum for *Gamma*-like viruses in felids, unveiling a frequent history of horizontal interorder transmissions among mammals. Interspecies transmission is suggested for the origin of RD114, ERV-DCs and FeLV [1,13,24]. The *Gamma*-like tree topology (Figure 3) indicated that the divergence between clusters (lineages) of felid elements reflects their origins from different mammal hosts and the different groups found within these lineages points to further diversification, after these viruses jumped into the Felidae. The split between the *Panthera* lineage and those of other moderns cats, estimated to have occurred *ca.* ten million years ago [16], suggests that the majority of retroviral lineages shown in this study have existed in a common ancestor at least since middle Miocene. Furthermore, LTR divergence of *Gamma*-like sequences provided support for considerably old times of ERV insertion in most cases, although some sequences seem to have integrated more recently into felid genomes. The insertions within lineages shown in Table 2 that dated after ten million years ago did not necessarily represent new infections of exogenous retroviruses such as FeLV, since they could also result from postintegration diversification. Moreover, the divergence time of several LTR sequences analysed here suggests that *Gamma*-like lineages may have integrated into common ancestors in periods close to/during the diversification of modern felids (Table 2). This may explain the monophyly of the eleven *Gamma*-like lineages from felids, which may be further scrutinized as additional genomes from this family and other mammals become available.

Although our data suggest that cats acquired *Gamma*-like retroviruses during multiple cross-species transmission events in the past, the exact origin of these proviruses cannot be strictly deduced based on current information. It is thought that humans became infected with HIV during butchery of primates hunted as bush meat [25]. Competition for ecological resources is also an important factor for cross-species transmission, as exemplified for FIV [26]. Felid ERVs are close to those of primates, rodents, carnivores and bats, all of which are common prey items of modern wild cats [27]. Thus, it is possible that cats also acquired retroviruses by hunting and/or scavenging carcasses. Although this hypothesis may be considered speculative, mainly because we cannot recover the real scenarios in which the ancestral cats lived or their exact trophic interactions, the phylogenies reconstructed herein are consistent with that hypothesis. Alternatively, felids could have been the source of transmission of some *Gamma*-like lineages. Primate and felid lineage IX grouped in a well-supported clade, with the felid lineage X as a basal clade (albeit poorly supported). Similarly, marine carnivores showed disconnected lineages I and II. To confirm the hypothesis of cross-transmission events as opposed to an older integration, it would be necessary to investigate insertion regions among the different mammalian genomes. The detection of orthologous sequences could indicate a more ancient event, considering that new insertions from infection result in random integrations.

The evolutionary tree in Figure 3 shows that some felid ERV lineages are very closely related to their sister groups, while some lineages are consistently more divergent. For example, it is noticeable that lineages III and VII probably arose from a single cross-species transmission event due to common ancestry with sequences from bats. However, the longer branches connecting lineage III with bat sequences as well as the long internal branch lengths within the felid clade suggest old infection events. Consistent with this, we estimated integration events having occurred before the basal divergence of modern felids (see Table 2), around ten million years ago [16]. On the other hand, the shorter branches splitting bat and felid sequences of lineage VII and the low genetic distances estimated among felid sequences suggest that this lineage arose more recently. Furthermore, the LTR divergence in the two ERV sequences of lineage VII (see Table 2) as well as in the ERV sequence found in the contig [GenBank: AAPE02061792.1] from the bat *Myotis lucifugus* (LTR K2P distance of 0.01, dating at 2.17 MY using the substitution rate of 2.3 E-9) supports the idea that these lineages have had recent activity.

## Conclusions

Our results strongly indicate that retroviruses have circulated in wild cats throughout their evolution, with the majority of the detected ERV clades having arisen during or prior to the basal diversification of modern felid lineages. We hypothesize, based on our phylogenetic inferences, that modern cat species descend from survivors of several retroviral infections: Pol similarities indicate that they comprised at least eleven lineages of gammaretroviruses (including class I ERVs), three of betaretroviruses (including class II ERVs) and one divergent group whose position in our phylogeny was difficult to establish confidently relative to known retroviruses. We also inferred that horizontal inter-ordinal transmission among mammals has been quite common, particularly among class I ERVs. Our findings can assist in future studies that aim to identify and characterize ERVs in mammalian genomes, and highlight the importance of further studies assessing the evolutionary history of ERVs in the genomic landscape of other carnivore species.

## Methods

### Samples

Samples of Brazilian wild cats were used to capture major components of the ERV diversity in Neotropical felids. They were originally obtained from road-killed individuals or wild animals captured from field ecology studies at the Laboratory of Genomics and Molecular Biology of Pontifícia Universidade Católica do Rio Grande do Sul (PUCRS), Brazil. A sample of *Leopardus geoffroyi* was donated by the Museum of Sciences and Technology of PUCRS*.* Eight species (one specimen each) of Brazilian wild cats were included in this study: southern tigrina Brazil (*Leopardus guttulus*; formerly part of *Leopardus tigrinus*), margay (*Leopardus wiedii*), ocelot (*Leopardus pardalis*), jaguarundi (*Puma yagouaroundi*), pampas’ cat (*Leopardus colocolo*), geoffroy’s cat (*Leopardus geoffroyi*), cougar (*Puma concolor*) and jaguar (*Panthera onca*). Genomic DNA was extracted from whole blood or tissues using PureLink Genomic DNA Kit (Life Technologies, Carlsbad, USA) according to the manufacturer’s protocol. To avoid cross contamination, samples were handled separately from each other during DNA extraction, PCR amplification, cloning and sequencing.

### PCR, cloning and sequencing

Four degenerate primers targeting a fragment of the protease-reverse transcriptase (*pro-pol*) genes were used for PCR amplification as previously described [28]. These viral genomic regions have been shown to be conserved, allowing the reconstruction of robust phylogenetic hypotheses [29,30]. Furthermore, the targeted regions allowed the comparison with previously published ERV sequences. PCRs were conducted with 2 min at 80°C followed by 35 cycles of denaturation at 94°C for 30s, annealing at 45°C to 50°C for 30s, and polymerization at 74°C for 60s, and one final cycle at 94°C for 30s, 45°C for 3 min, and 74°C for 10 min. Reaction conditions were performed with 50 mM KCl, 10 mM Tris-HCl (pH 8.3), 1.5 mM MgCl_2_, 200 mM dNTPs, 150 pmol of each primer, 100 ng of template DNA, and 2 U of Platinum Taq polymerase (Invitrogen, São Paulo, Brazil).

PCR-amplified DNA products were electrophoresed into 1% low melting agarose gels stained with ethidium bromide. Products ranging between 600 and 1200 bp were excised from the gel and purified with the Wizard SV gel kit (Promega, Madison, USA). DNA fragments were then cloned into pGEM-T Easy (Promega) and transfected into *E. coli* JM109 cells, following the manufacturer’s recommendations. Colony plasmid miniprep DNA purification was performed with the method of lysis by alkali [31] or with the Wizard Plus SV Miniprep DNA Purification System kit (Promega). Sequencing of purified PCR products was performed with the BigDye Terminator kit v.3.1 (Life Technologies, Carlsbad, USA), using the universal primers T7 and SP6, and reactions were run on an ABI-PRISM 3100 Genetic Analyzer (Life Technologies) following the manufacturer’s protocol. A total of 236 clones comprising the eight felid species were evaluated, corresponding to an average of 31 clones (25- 40) for each species studied.

To confirm the results of degenerate PCR we designed lineage-specific primers (see Additional file 4: Figure S2) and performed PCR using aliquots of genomic DNA of three specimens of each Brazilian wild cat species. The expected products were around 200 bp for the *Gamma*-like lineage and around 400 bp for the LwiJO7007 group. Selected primers were subjected to *in silico* PCR searches through the UCSC In-Silico PCR (http://genome.ucsc.edu) using the *Felis catus* (ICGSC Felis_catus 6.2/felCat5; Sep. 2011) genome assembly. Additionally, PCR products were sequenced in order to confirm their specificity.

### Sequence nomenclature and lineage definition

The ERV sequences obtained by PCR were named as follows: the first three letters correspond to the species’ name, while the latter two represent the primer used in the PCR; the numbers correspond to the size of the fragment followed by the clone number. For example: Lwi (*Leopardus wiedii*) JO (primer) 700 (fragment size) 7 (clone number). The retroviral sequences obtained in this study have been deposited in GenBank (accession numbers KJ632899-KJ632941 and KJ650005). The sequences Lg_EM_1000_3 [GenBank: JX406427] from *L. geoffroyi*, Pco_JO_1000_6 [GenBank: JX406428]) and Pco_JO_1200_1 [GenBank: JX406429] from *P. concolor* were published in a previous study [32]. Sequences retrieved from GenBank and used in our phylogenetic analyses are listed in Additional file 1: Table S1.

A group of sequences identified in felids and closely related to class I ERVs (which includes gammaretroviruses) was defined as the *Gamma*-like lineage. Within this group, lineage classification was based on phylogenetic analyses and specifically delimited by host switches between Felidae and other mammalian groups.

### Data mining and alignments

DNA sequences obtained by PCR in this study were compared to GenBank entries using the program Blastn to identify ERV sequences among the amplified retroelements. Additionally, we performed specific searches using the program *RepeatMasker* via the UCSC Genome Database (https://genome.ucsc.edu) and the program *Censor* [18]. Database searches were performed online from April to September 2013.

We performed extensive phylogenetic analyses of the identified ERVs along with GenBank-retrieved sequences, using four different datasets constructed with distinct strategies. Dataset 1 (DS1) was the broadest, and aimed to maximize the number of included ERV sequences by focusing only on the RT fragment, which is more conserved, allowing the comparison of amino acid data among very divergent different retroviral genera. Dataset 2 (DS2) focused specifically on the group of 19 sequences that were most similar to our LwiJO7007 element, based on Blast searches against different databases. We analyzed only the segment contained in this ERV that could be reliably aligned to these other sequences (using the alignment strategy described below), i.e. 613 bp (93% of the original positions), of which 441 represented the RT fragment. Also, since we observed no DNA saturation in this dataset (given the more shallow sampled time span than DS1, and the conserved nature of the segment), we used nucleotides instead amino acids for this analysis.

For datasets 3 and 4 (DS3 and DS4), we maximized the number of molecular characters (amino acids) by using the *pro/pol* fragment and analysing divergent groups separately. DS3 focused on the *Gamma*-like elements (described in detail below), while DS4 focused on the class II ERVs (incorporating GenBank sequences retrieved with Blast searches, as well as elements previously reported in the literature).

To construct DS3, which comprised most of the sequences identified in this study, we initially defined putative felid *Gamma*-like groups through phylogenetic analysis using the neighbour-joining (NJ) algorithm implemented in MEGA 5 [33]. The alignment used was 327 amino acids long and contained 39 sequences, revealing the existence of eight different ERV lineages in this dataset (with > 15% divergence between them). To further characterize these groups, we carried out MegaBlast searches out using one representative sequence of each lineage as a query against the whole genome shotgun (WGS) contigs from *Felis catus* publicly available. Furthermore, tBlastn searches in the nucleotide collection (nr/nt) and WGS databases were performed using the NCBI Blast suite (http://blast.ncbi.nlm.nih.gov/). Initially, we selected all sequences retrieved in the Blast results, removing those with less than 30% identity and 70% coverage, presenting an *e*-value > 0.001, identical sequences and those with large indels. Short interspersed elements (SINEs) inserted within ERV sequences were excluded from the alignments. Alignments composed of sequences retrieved both from the nr/nt and the WGS databases were merged to create DS3, removing very similar sequences (≥95% identity) belonging to the same species. To reduce computer time, only one or two sequences per host species were kept in the final alignment for each lineage. However, as our focus was on felids, the majority sequences of the domestic cat were kept. Overall, this effort resulted in the identification of three additional lineages contained in this *Gamma*-like group, making up a total of 11 lineages contained in DS3.

During the course of this study, the genome sequence from *Panthera tigris altaica* was released in NCBI. We performed a second round of Megablast searches against this genome, using one or two representatives of the 11 lineages described above as queries. The highest hits belonging to *Panthera tigris altaica* (all with *e*-values close to zero), covering at least 70% of the query sequences, were incorporated into DS3.

Amino acid alignments were constructed for DS1, DS3 and DS4 as described in [10], using as template the known amino acid sequences of previously described retroviruses and virtual translations of novel retroviral sequences that do not have nonsense mutations in the amplified region. Sequences were carefully edited and manually adjusted. Although many sequences contained nonsense and/or frameshift mutations, we considered only their full coding capacity by eliminating these mutational events.

In the final alignments, the deduced fragments of virus-like Pro/Pol proteins from *Gamma*-like sequences were aligned again using Mafft v.7.017 [34] with the FTT-NS-2 option; the L-INS-I option was used to align RT sequences related to class II ERVs and nucleotide sequences related to LwiJO7007. Regions of high sequence diversity and hence uncertain alignment were also eliminated using Gblock v.0.91 [35] with the following options: minimum length of block of 5, gap allowed with half (treated as gaps positions where 50% or more sequences have a gap), minimum number of sequences for a flanking position of 120 for DS1, 15 for DS2, 197 for DS3, 12 for DS4 and maximum number of contiguous non-conserved position of 10 for DS3 and of 8 for DS1, DS2 and DS4. DS1 consisted of 152 sequences and 147 amino acid residues (56% of the original positions). DS3 consisted of 263 sequences, including 198 amino acid residues (50% of the original positions). DS4 comprised 19 sequences and 236 amino acid residues (68% of the original positions).

### Dating ERV insertions using long terminal repeats (LTR)

To estimate the age of felid ERVs, we focused on *Gamma*-like ERV lineages established in this study, estimating the age of proviral *pro/pol* sequences found in the domestic cat and tiger genomes that were related to the elements of Neotropical wild cats. Our analyses were based on nucleotide divergence between 5′- and 3′-LTR sequences at the same insert, assuming that all differences arising post-integration evolved neutrally [36]. The analysis was based on the relation T = (D/R)/2, where T is the time of integration, D is the divergence between the LTRs and R is the nucleotide substitution rate. We estimated D using Kimura’s two-parameter (K2P) model [37] with MEGA5. Because the LTR mutation rates of from felid ERVs are unknown, we employed three alternative substitution rates reported in the literature: 2.3E-9 and 5E-9 substitutions per site per year, based on human ERVs [38], and 1.2E-8 substitutions per site per year, a general rate estimated for felid nuclear DNA [39] and previously used for domestic cat ERVs [13]. The LTR sequence divergence in contigs that contained LTRs flanking putative proviral *Gammaretrovirus* sequences (~7-11 kb) was assessed. Typical LTR features such as the presence of conserved motifs corresponding to (i) short inverted repeat motifs TG and CA at both LTR ends, (ii) the polyadenylation signal (AATAAA motif) and (iii) a less conserved AT-rich stretch corresponding to the TATA box [40] were also examined. Furthermore, potential amino acid sequences of *gag*-*pol-env* genes were searched using Blastx and manually assessed to verify the structural organization of these genes within the putative ERV sequence. Only sequences that presented matches with high statistical significance (i.e. *e*–values ≤ 0.001) were analyzed. For each lineage identified in our *Gamma*-like phylogeny (DS3), at least one representative in which the conditions described above were present was selected. Therefore, the representative was a putative full-length ERV delimited by the identification of retroviral *gag*, *pol* and *env* sequences positioned next to each other and placed between 5′- and 3′-LTRs. We were then able to estimate the time of the insertion of that ERV sequence and further compared it with temporal inferences of felid speciation times described in the literature.

### Phylogenetic analyses

A phylogenetic approach was used to assess the evolutionary relationship among ERV sequences found by both PCR and *in silico* analyses. Maximum likelihood (ML) trees were performed using Randomized Accelerated Maximum Likelihood conducted with RAxML v.7.2.6 [41] using the raxml GUI 1.3 interface [42] and performing 1,000 bootstrap replicates to evaluate branch support. The best substitution models were estimated using jModeltest v.0.1.1 [43] for the nucleotide dataset and ProtTest v.2.4 [44] for the amino acid datasets, both employing the Akaike information criterion (AIC). The best-fit substitution models selected were as follows: JTT + G (DS1 and DS3); RtREV + I + G (DS4); and GTR + G (DS2).

## Additional files

Additional file 1: Table S1. Sequences included in the molecular phylogenetic analyses along with GenBank accession numbers and, in case of WGS datasets, the matched positions in the Blastn searches. Additional file 2: Figure S1. ML tree topology showing evolutionary relationship of tiger and domestic cat ERVs from Dataset 2 and DNA sequences from Brazilian wild cats obtained by LwiJO7007 group specific PCR. Sequences from felids shown in Figure 2 are indicated in bold. *Felis catus* (chrB:182483770-182484196) is from *in-silico* PCR. Bootstrap values > 70% indicated next to their respective nodes were assessed using 1000 replications. The tree was based on 390 bp under the GTR model and was rooted by the sequence from tiger [GenBank: ATCQ01106762] according to Figure 2. Additional file 3: Table S2. Results of the nucleotide Blast analysis of sequences from the *Gamma*-like lineages (see Additional file 4) amplified by lineage-specific PCR. Additional file 4: Figure S2. Detection of *Gamma*-like endogenous retrovirus in eight species of Felidae by lineage-specific PCR amplification. Lineages I - IV and VII - XI are those indicated in Figure 3. The primer sequences were designed based on sequence homology among ERVs of each lineage and are shown on the top of each gel picture. Three individual specimens for each species were amplified and shown by gel electrophoresis analysis.
