# Supplementary figures and images for: Identification and characterization of diverse groups of endogenous retroviruses in felids
Source: Retrovirology. 2015 Mar 15;12:26. doi: 10.1186/s12977-015-0152-x (PMC4373062; doi:10.1186/s12977-015-0152-x)

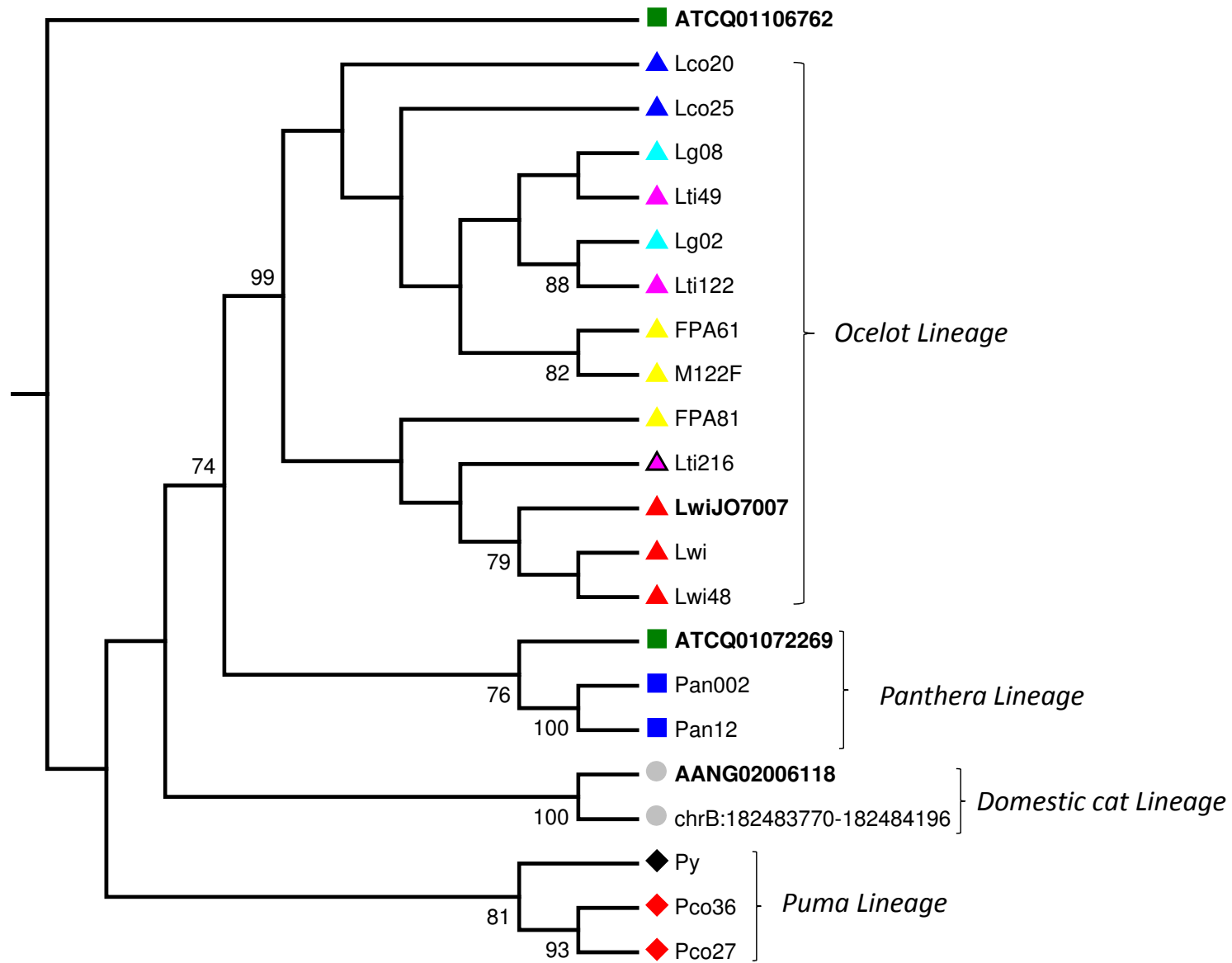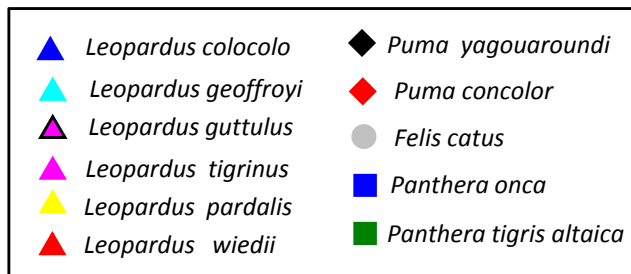

Supplement: Additional file 2: Figure S1. — ML tree topology showing evolutionary relationship of tiger and domestic cat ERVs from Dataset 2 and DNA sequences from Brazilian wild cats obtained by LwiJO7007 group specific PCR. Sequences from felids shown in Figure 2 are indicated in bold. Felis catus (chrB:182483770-182484196) is from in-silico PCR. Bootstrap values > 70% indicated next to their respective nodes were assessed using 1000 replications. The tree was based on 390 bp under the GTR model and was rooted by the sequence from tiger [GenBank: ATCQ01106762] according to Figure 2. [file 12977_2015_152_MOESM2_ESM.pdf]
