# Supplementary material for: Identification and characterization of diverse groups of endogenous retroviruses in felids
Source: Retrovirology. 2015 Mar 15;12:26. doi: 10.1186/s12977-015-0152-x (PMC4373062; doi:10.1186/s12977-015-0152-x)
Supplement: Additional file 4: Figure S2. — Detection of Gamma-like endogenous retrovirus in eight species of Felidae by lineage-specific PCR amplification. Lineages I - IV and VII - XI are those indicated in Figure 3. The primer sequences were designed based on sequence homology among ERVs of each lineage and are shown on the top of each gel picture. Three individual specimens for each species were amplified and shown by gel electrophoresis analysis. [file 12977_2015_152_MOESM4_ESM.pdf]

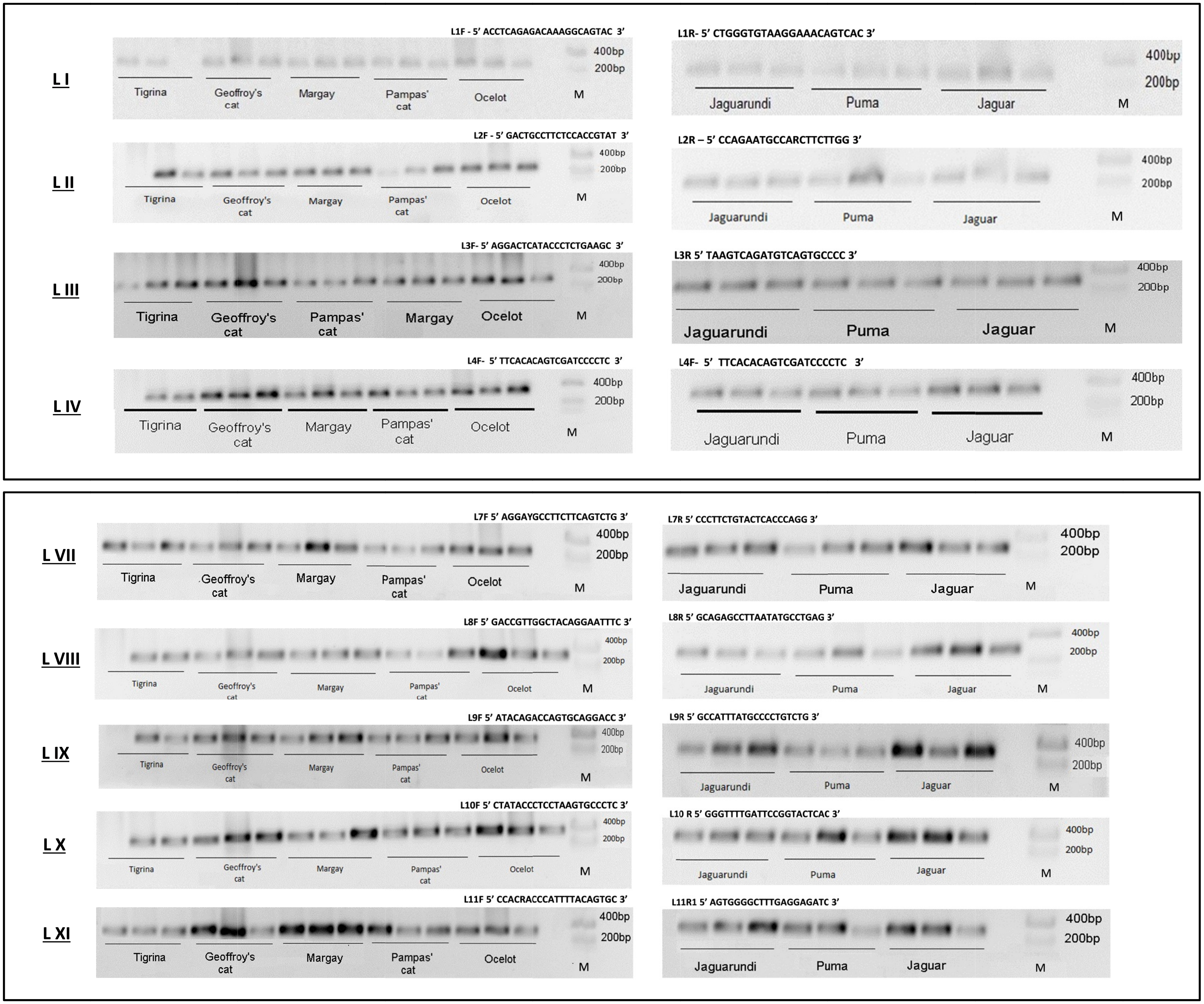

L I
L1F - 5' ACCTCAGAGACAAAGGCAGTAC 3'
400bp
200bp
Tigrina
Geoffroy's cat
Margay
Pampas' cat
Ocelot
M
L1R- 5' CTGGGTGTAAGGAAACAGTCAC 3'
Jaguarundi
Puma
Jaguar
L II
L2F - 5' GACTGCCTTCTCCACCGTAT 3'
L2R – 5' CCAGAATGCCARCTTCTTGG 3'
L III
L3F- 5' AGGACTCATACCCTCTGAAGC 3'
L3R 5' TAAGTCAGATGTCAGTGCCCC 3'
L IV
L4F- 5' TTCACACAGTCGATCCCCTC 3'
L4F- 5' TTCACACAGTCGATCCCCTC 3'
L VII
L7F 5' AGGAYGCCTTCTTCAGTCTG 3'
L7R 5' CCCTTCTGTACTCACCCAGG 3'
L VIII
L8F 5' GACCGTTGGCTACAGGAATTTC 3'
L8R 5' GCAGAGCCTTAATATGCCTGAG 3'
L IX
L9F 5' ATACAGACCAGTGCAGGACC 3'
L9R 5' GCCATTTATGCCCCTGTCTG 3'
L X
L10F 5' CTATACCCTCCTAAGTGCCCTC 3'
L10 R 5' GGGTTTTGATTCCGGTACTCAC 3'
L XI
L11F 5' CCACRACCCATTTTACAGTGC 3'
L11R1 5' AGTGGGGCTTTGAGGAGATC 3'
